# Trabectedin-Related Heart Failure: Case Report and a Systematic Review of the Literature

**DOI:** 10.3389/fonc.2021.694620

**Published:** 2021-11-18

**Authors:** Julien Catherine, Christiane Jungels, Valerie Durieux, Coralie Deliens, Bogdan Grigoriu

**Affiliations:** ^1^ Unité de Soins Intensifs et Urgences Oncologiques, Service de Médecine Interne, Institut Jules Bordet, Université Libre de Bruxelles (ULB), Bruxelles, Belgium; ^2^ Département de Médecine Oncologique, Institut Jules Bordet, Université Libre de Bruxelles, Bruxelles, Belgium; ^3^ Bibliothèque des Sciences de la Santé, Université Libre de Bruxelles (ULB), Bruxelles, Belgium; ^4^ Laboratoire de Médecine Factuelle, Faculté de Médecine, Université Libre de Bruxelles (ULB), Bruxelles, Belgium; ^5^ Pharmacie Hospitalière, Institut Jules Bordet, Université Libre de Bruxelles (ULB), Bruxelles, Belgium

**Keywords:** trabectedin, toxicity, acute, cardiac, drug-induced, cardiac biomarkers

## Abstract

New drugs come not only with benefits but also with unexpected toxicities which need to be promptly recognized and managed. Starting from a scholar case of acute heart failure with preserved ejection fraction following the administration of trabectedin (ET-743, Yondelis^®^) in a patient with a metastatic solitary fibrous tumor, we performed a systematic review of the literature encompassing the results of previous cardiac safety analysis published ten years ago, a review of clinical trials published during the last 10 years as well as single-case descriptions related to trabectedin cardiotoxicity. The estimated incidence of cardiac toxicity was 3,4% among patients receiving trabectedin, with recent data suggesting a higher rate of heart failure than previously recognized. Previous or concomitant anthracyclines exposure may represent a risk factor. Assaying for NT-pro-BNP may be useful for the early detection of individuals with trabectedin-induced heart failure.

## Introduction

Trabectedin (code ATC L01CX01, also known as ecteinascidin 743 or ET-743 – Yondelis ^®^) is a synthetically produced tetrahydroisoquinoline alkaloid originally derived from the Caribbean marine tunicate, Ecteinascidia turbinata (1). Trabectedin (Tbt) has a direct effect against tumor cells but has also host-modulating properties (2–4). The drug interacts with the DNA minor groove and alkylates guanine at the N2 position, which bends towards the major groove. It is thought that the molecule affects several transcription factors involved in cell proliferation, particularly through the transcription-coupled nucleotide excision repair system (3). Tbt blocks the cell cycle at the G(2) phase, cells in the G(1) phase being most sensitive to the drug. It also inhibits overexpression of the multidrug resistance-1 gene (MDR-1) coding for the P-glycoprotein (1). Tbt is approved for the treatment of advanced soft tissue sarcomas (STS) after failure of treatments with anthracyclines and ifosfamide or in patients who are unable to tolerate such agents (5). It is also approved in combination with pegylated liposomal doxorubicin for advanced, relapsed ovarian cancer.

The most common adverse events associated with Tbt are myelosuppression, digestive toxicity (anorexia, nausea, vomiting, diarrhea or constipation), general condition decline and hepatic toxicity (hyperbilirubinemia, elevation of alanine aminotransferase or/and aspartate aminotransferase) (6). The approved summary of product characteristics mentions the need to monitor left ventricular ejection fraction (LVEF) before initiation and after treatment with Tbt, and recommend that an absolute decrease of LVEF ≥15%, or ≥5% if less than the lower limit of normal, should prompt drug discontinuation. These toxicities are believed to be present only in patients with ovarian cancer receiving previous or concomitant anthracyclines (7). Acute cardiac dysfunction is not mentioned.

We report a case of acute cardiac toxicity which is not classically acknowledged and infrequently described during early phases of drug development. A systematic review of the literature addressing Tbt-associated cardiac adverse effects (CAE) is presented.

## Case Report

A 53-year-old man with a metastatic solitary fibrous tumor (SFT) was admitted because of recent onset dyspnea. Two days before admission and one day after a third Tbt cycle, he presented abrupt shortness of breath with orthopnea and ankle swelling without chest pain, fever or chills. The same symptoms have already been present after the previous Tbt administrations with increased severity with each cycle, but resolved spontaneously after five to seven days.

He suffered from arterial hypertension, hypercholesterolemia, type 2 diabetes and hypothyroidism. He had been diagnosed with a cerebral SFT ten years earlier in the setting of seizures, revealing a right occipital mass which was surgically removed immediately followed by whole brain irradiation. Five years later, hepatic and pancreatic relapses were managed by surgical resection. One year later, liver and lung progression mandated weekly adriamycin (cumulative dose 375 mg/m^2^), with disease control for 9 months. He then received a combination of ramucirumab and an angiopoietin 2 inhibitor (phase I trial), discontinued after 4 months for grade III hypertension. After 4 months of therapeutic break he received pazopanib for metastatic progression. After a new multi-site metastatic progression, a fourth line of cyclophosphamide (200 mg daily) and a fifth line of paclitaxel (80 mg/m^2^ weekly) were administered, both without anti-tumoral effect. Three months before the current admission Tbt 1.5 mg/m^2^ every three weeks was initiated for disease progression.

Upon hospital admission, physical examination revealed tachypnea, bilateral jugular veins distention and inferior limb edema with crackles in the left lower lung area. Arterial blood gases, breathing room air, showed pH 7,47; PaCO_2_ 33 mmHg; PaO_2_ 68 mmHg and blood analysis a grade 2 anemia, grade 1 acute renal failure and NT-pro-BNP at 2800 pg/mL (N < 143 pg/mL). D-dimers and troponin were within normal range while EKG showed only a left atrial hypertrophy. An angiographic chest CT revealed already known bilateral pulmonary metastases and no arguments for any infectious process, pleural or pericardial effusion. NT-pro-BNP measurements gradually increased since receiving Tbt (from 356 pg/mL after the first cycle to 2800 pg/mL four days after the third cycle). A drug-related cardiac failure was suspected and intravenous furosemide. Intravenous furosemide (40 mg) resulted in rapid clinical improvement. Four days later an echocardiography was unremarkable. Subsequent coronary computed tomographic angiography and an isotopic evaluation of left ventricular ejection fraction were normal. He was discharged with oral furosemide with symptoms relief. Tbt was permanently discontinued.

## Literature Review

### Methodology

The literature search was performed by a scientific librarian (VD) experienced in searching for medical and scientific publications and by a physician (BG) trained in evidence-based medicine. Ovid Medline (*via* OvidSP interface) and SciVerse Scopus databases were searched in June 2020. The “PICO” (population, intervention, comparator, outcome) model for clinical questions was used to identify the concepts included in the questions. The corresponding search criteria of “P” and “I” were translated into MeSH terms, and free-text keywords that were searched for in titles, abstracts, keywords and name of substances (**Appendix 1**). Identified manuscripts were divided into three categories: (1) previously available literature reviews, (2) a pooled analysis of data from clinical trials published between January 2010 and June 2020 and reporting the frequency of CAE, and (3) all clinical studies reporting detailed information about the characteristics of CAE (including clinical trials, case series or reports). In addition, a Vigibase search was performed.

### Previous Literature Reviews

In 2011, Lebedinsky et al. (8) published a comprehensive cardiac safety analysis compiling CAE associated with Tbt administration in phase I to III clinical trials, pharmacovigilance signaling and spontaneously reported cases available at that time. Phase I trials evaluating Tbt alone or with doxorubicin or pegylated liposomal doxorubicin (PLD) revealed a safe cardiac profile, with only six patients experimenting CAE, including three grade 4 toxicity (one cardiac arrest and two grade 4 atrial fibrillation) among 438 patients. Among 155 patients receiving a combination of Tbt and anthracyclines in phase I trials, a left ventricular ejection fraction (LVEF) alteration was recorded in 5 patients but resolved after PLD discontinuation in three patients who continued Tbt treatment (no follow-up available for the remaining two patients). In a retrospective pooled analysis by Le Cesne (9) gathering 1,132 patients with solid tumors treated in phase II clinical trials with Tbt as single agent, CAE of any grade occurred in twenty patients, of which three were grade 3 or 4 - one grade 3 atrial fibrillation (AF) and two (grade 3 and 4) heart failures (HF). The most common CAE were tachycardia and palpitations. Finally, Lebedinsky et al. reported a post-marketing experience with Tbt as single agent in STS consisting of 4 cases of CAE (2 cardiac arrests and 2 heart failures) among 2046 patients (8).

### Polled Analysis of Clinical Trial Data

To complete the cardiac safety analysis of Lebedinsky et al., we initially reviewed all clinical trials testing Tbt alone or in combination with other agents published between 2010 and 2020. Among the 36 clinical trials retrieved from the literature during this period, CAE were reported in 13 publications, mostly phase II trials, regrouping 1061 patients, which are detailed in **Table 1**. The mean incidence of cardiovascular adverse events was 3,4% (36/1061). In contrast with previous findings, palpitations and tachycardia were as frequent as HF, with arrhythmia and/or EKG modifications accounting for 11/36 cases and HF for 11/36 cases. Among the former group, 3 cases of atrial fibrillation (AF) (any grade) were highlighted and QT interval prolongation accounted for 5 cases. Concerning HF, grading wasn’t available in 7 patients, the 4 remaining cases being G3/G4. Interestingly, 6/12 cases occurred in the phase III OVA-301 trial which compared combined Tbt and PLD with PLD alone in patients with recurrent ovarian cancer previously treated with a platinum-based regimen. In the PLD arm, a single case of HF was reported, suggesting a possible effect of combination therapy. No further details were available concerning the clinical presentation, number of cycles administered before CAE occurrence or the prognosis. The occurrence of LVEF reduction (any grade) was similar between the two arms (<3%). Among the other trials, only 3 cases of LVEF reduction were reported but there was no systematic LVEF monitoring. Myocardial infarction occurred in one patient receiving Tbt as monotherapy (23). Arterial hypertension occurred in 3 patients in a trial evaluating a combination of Tbt and olaparib, the latter not being described associated with such risk (24). A search in the WHO VigiBase of individual case safety reports database found, as of 13 August 2020, a total of 38 cases while a statistical simulation predicted an expected number of 5 cases only (25).

**Table 1:** Clinical trials using trabectedin alone or in combination with other agents and reporting cardiac adverse effects.

1^st^ author, year of publication	Cancer type	Study design	Incidence of CAE	Hypertension		Arrythmia/EKG abnormalities		Myocardial infarction		Heart failure		TTE abnormalities	Comments
				G1-2	G3-4	G1-2	G3-4	G1-2	G3-4	G1-2	G3-4		
Monk, 2010 (10)	Ovarian	Phase III, Tbt + PDL *vs* PLD	6/337 (Tbt+PLD arm)	–	–	–	–	–	–	6		7/337 LVEF reduction, not graded	
Monk, 2011 (11)	Ovarian	Phase II, Tbt+docetaxel	3/71	–	–	–	–	–	–	–	–	–	3 grade I CAE not described
Michaelson, 2012 (12)	Prostate	Phase II, Tbt alone	3/58	–	–	–	1	–	1	–	1	–	
Sessa, 2013 (13)	Solid tumors	Phase I, Tbt alone, dose esc.	3/12	–	–	1	1	–	–	–	–	1 grade 1 or 2 LVEF reduction	
Delaloge, 2014 (14)	Breast	Phase II, Tbt alone	2/38	–	–	–	–	–	–	–	1	1 LVEF reduction, not graded	
Ueda, 2014 (15)	Sarcoma	Phase I, Tbt dose esc.	3/15	–	–	2	1	–	–	–	–	–	3 QT interval prolongation
Bui-Nguyen, 2015 (16)	Sarcoma	Phase IIb, Doxo *vs* Tbt over 3h *vs* Tbt over 24h	3/90 (Tbt arm)	–	–	–	–	–	–	–	–	–	3 grade 3 or 4 CAE, not described
Pautier, 2015 (17)	Sarcoma	Phase II, Tbt+Doxo	1/108	–	–	1	–	–	–	–	–	–	
Kawai, 2015 (18)	Sarcoma	Phase II, Tbt *vs* BSC	2/39 (Tbt arm)	–	–	1	1	–	–	–	–	–	2 QT interval pronlongation
Le Cesne, 2015 (19)	Sarcoma	Phase II, Tbt alone interruption *vs* continuation arm	2/46	–	–	–	1	–	–	1		–	HF occurred in continuation arm
Gaducci, 2018 (20)	Sarcoma	Phase II, Tbt alone	2/126	–	–	1	–	–	–	–	1	–	
Grignani, 2018 (21)	Sarcoma	Phase Ib, Tbt+olaparib	3/50	1	2	–	–	–	–	–	–	–	
Colombo, 2019 (22)	Ovarian	Phase II, Tbt + bevacizumab +/- carboplatin	2/71	–	–	–	–	–	–	–	1	1 grade 3 LVEF reduction	Both cases occurred in patients receiving the 3 agents
		Total	36/1061	1	2	6	5	0	2	11			

BSC, best supportive care; Dose esc., dose escalation; Doxo, doxorubicin; LVEF, left ventricular heart failure; PLD, pegylated liposomal doxorubicin; Tbt, Trabectedin; TTE, transthoracic echocardiography; vs\, versus. “-” Not reported.

### Clinical Characteristics of Trabectedin-Associated Cardiotoxicity

A review of clinical studies, case reports and small case series was performed to gain insight into the clinical pattern of Tbt-induced CAE (**Table 2**). Thirteen cases were found in the literature. The incidence of CAE varied between 0.7% and 6.7%. Heart failure was the most frequent reported CAE occurring in 7 patients with a median age of 70 years (range 58 to 81 years) after a median number of 4,7 administrations of Tbt (range 2 to 7), and all of them had received previous chemotherapy. Pulmonary edema was the most common clinical presentation (6/7) and the evolution was mainly not fatal (6/7). The presence of pre-existing cardiovascular risk factors was reported in one patient but the information was lacking in most cases. In 4 cases, no other cause was suspected by the authors.

**Table 2:** Detailed analysis of single cases of trabectedin-induced cardiotoxicity reported in clinical trials, case series and case reports.

1^st^ author, year of publication	Study design	Nb of subjects	Incidence	Indication	Sex, age	Nb of cycles*	CV risk factors	Previous chemotherapy	Presentation	Evolution	Other cause suspected
Krasner, 2007 (34)	Phase II trial, Tbt alone	147	1/147	Ovarian cancer	F, 58y	7^th^	NM	Yes, Carboplatin Taxane	Grade 3 dyspnea, pulmonary edema, central chest pain, bilateral pleural effusion, pulmonary hypertension, and left cardiac failure	Fatal	No
Messersmith, 2008 (35)	Phase I trial, Tbt + gemcitabine	15	1/15	Solid tumor	NM	NM	NM	Yes, NM	Congestive heart failure and pulmonary edema	Not fatal	NM
Ferrandina, 2013 (36)	Retrospective, multicenter, Tbt alone	98	2/98	Ovarian cancer	F, > 70y	NM	Yes	Yes, Anthracyclines Taxane	Grade 3 arrythmia	Not fatal	NM
					F, > 70y	NM	Yes	Yes, Anthracyclines Taxane	Myocardial infarction	Not fatal	NM
Moriceau, 2015 (37)	Retrospective, monocenter, Tbt alone	59	2/59	Soft tissue sarcomas	NM	NM	NM	Yes, NM	Acute cardiac failure, pulmonary edema	Not fatal	Sodium overload, pulmonary embolism
					NM	NM	NM	Yes, NM	Acute cardiac failure, pulmonary edema	Not fatal	None
Blum, 2016 (38)	Phase II trial, Tbt alone	87	1/87	Breast cancer	F, NM	NM	Yes	Yes, NM	Congestive heart failure	Not fatal	Presumed
Doherty, 2019 (39)	Case series about cardiac toxicity, monocenter, Tbt alone	6	–	Uterine myosarcoma	F, 71y	5^th^	None	Yes, Anthracyclines	Pulmonary edema, myocardial infarction	Not fatal	No
				Spindle cell and pleomorphic sarcoma of thigh	F, 81y	2^nd^	Yes (AF, ventricular pacing)	NM (no anthracyclines)	Pulmonary edema	Not fatal	No
				Carcinosarcoma	F, 72y	3^rd^	None	Yes, Anthracyclines	Myocardial infarction	Not fatal	No
				Spindle cell and pleomorphic sarcoma of thigh	F, 68y	1^st^	None	Yes, Anthracyclines	Cardiac arrest	Fatal	No
				Diaphragmatic leiomyosarcoma	M, 75y	2^nd^	Yes (AF)	Yes, Anthracyclines	Fast atrial fibrillation	Fatal	Yes, Lesion abutting heart may have predisposed as well as pneumonia
				Vulval leiomyosarcoma	F, 73y	2^nd^	None	Yes, Anthracyclines	Fast atrial fibrillation	Not fatal	Lesion abutting heart may have predisposed

*Number of trabectedin cycles administered before cardiac adverse event onset.

AF, atrial fibrillation; F, female; M, male; Nb, number; NM, not mentioned; y, year.

Myocardial infarction occurred in three patients. In all cases, patients had received anthracyclines in their past history, age was between (71-72 years and one of them presented an acute HF (pulmonary edema) but no fatal issue occurred. In two cases, no other causative mechanism was suspected and Tbt-associated cardiac ischemia was suggested as mechanism.

Finally, three patients presented with cardiac arrhythmia including 2 patients with AF and one with a grade 3 arrhythmia not otherwise specified. Both patients with AF had neoplastic lesions abutting the heart which may predispose to arrhythmia and one patient had a concurrent infection as well as prior AF history, potentially explaining AF recurrence.

Overall, among these thirteen patients, three died from their CAE, including one patient with acute HF and one patient with fast AF. One patient had a cardiac arrest after an acute chest pain on day 10 of the first Tbt cycle. He suffered from arterial hypertension, statin-treated hypercholesterolemia, deep vein thrombosis with ongoing therapeutic coagulation and had previously received liposomal doxorubicin. A cardiac cause was suspected rather than a venous thrombo-embolic event given the therapeutic anticoagulation.

## Discussion

We report here a case of acute heart failure following Tbt administration in a heavily pretreated man with a SFT. Despite major cardiovascular risk factors, the work-up allowed us to exclude the most frequent etiologies of acute HF. The combination of acute NT-pro-BNP elevation concomitant with chemotherapy course, recurrence with increased clinical severity with each cycle, the exclusion of other causative conditions and ultimately the favorable evolution suggested the diagnosis of Tbt-induced HF. Acute heart failure has not been sufficiently recognized during initial phases of drug development and in practice, more than 3-4 Tbt cycles are given before such a potential severe side effect is recognized and assigned to the drug.

The emergence of new therapeutic options is a windfall for patients facing rare neoplasms. These advances go hand in hand with a prolonged survival which prompt clinicians to prevent or at least reduce long term drug-induced toxicity. However, ageing and the increase in the prevalence of cardiovascular risk factors face us to a population with high cardiovascular risk, precipitating the acute or delayed adverse cardiovascular effects of antineoplastic agents.

Chemotherapy-induced cardiac toxicity is a well-known phenomenon and can be divided into two major types. Type 1 cardiac toxicity is irreversible and is often dose dependent. Anthracyclines are typically associated with this kind of toxicity with an incidence of left ventricular dysfunction ranging from 3 to 5% with a cumulative doxorubicin dose of 400 mg/m2 to 18 to 48% for 700 mg/m2 (26). Type 2 chemotherapy-induced cardiac dysfunction is reversible and is not dose-dependent in most cases. Examples include the effect of monoclonal antibodies as trastuzumab with an incidence varying from 1,7 to 20,1% (26). The emerging cardiotoxic effects associated with immune checkpoint inhibitors are distinct from these toxicities and will probably lead to the individualization of a third type of toxicity characterized by its unpredictable occurrence and early and rapidly evolving pattern (e.g. fatal acute myocarditis) (27).

Recently, Nadruz et al. compared the characteristics of patients with HF induced by anti-neoplastic therapies (most of them had received anthracyclines, anthracyclines and radiotherapy (RT) or RT alone; 25% had breast cancer) and patients with HF unrelated to cancer (28). Cancer patients were younger, had less cardiovascular comorbidities, higher LVEF and a worst left ventricular diastolic function which was associated with a more severe outcome. Impairment in global longitudinal strain, functional capacity and ventilatory efficiency was similar between groups (28).

During its preclinical development, Tbt was not associated with relevant macroscopic, microscopic or functional cardiac alterations (8) and a first cardiac safety analysis showed a low incidence of cardiac adverse events (ranging between 0,2 to 3,3%), the most frequently reported being arrhythmia (most of cases experiencing palpitations and sinus tachycardia) (8). In our updated systematic review encompassing all published trials between 2010 and 2020, the incidence of CAE was similar (3,4%) but HF was as frequent as arrhythmia (including QT interval prolongation). Interestingly, 6/12 HF cases were reported in patients receiving Tbt/PLD combination while patients receiving PLD alone had a lower HF incidence (only 1/335). This observation suggests that Tbt could confer a higher risk of HF in patients receiving anthracyclines concomitantly.

Detailed analysis of single cases of Tbt-induced cardiotoxicity was in agreement with our previous findings, with HF representing the most commonly reported picture. Prior anthracyclines treatment, initial presentation of acute pulmonary edema and the non-fatal issue, are characteristics shared by most cases reported. Our patient was younger but had more cardiovascular risk factors than those previously reported. Lebedinsky et al. suggested that age > 70 years, previous anthracycline or cardiac radiotherapy exposure and cardiovascular risk factors (including hypertension, hypercholesterolemia, obesity and type II diabetes) may represent potential risk factors for CAE occurrence. Even if suggested, female gender cannot be included in this list given the overrepresentation of women in trials in which Tbt was evaluated (ovarian and breast cancer, uterine leiomyosarcoma).

Finally, our case raises the question of the utility of monitoring cardiac biomarkers which could identify cardiotoxicity earlier and allow prompt management. Numerous studies have evaluated available cardiac biomarkers, mainly troponins and natriuretic peptides (29, 30). An early prospective study assessed the prognostic value of troponin I (TnI) in a population receiving high dose chemotherapy with anthracyclines and/or alkylating agents and identified three groups: patients without any TnI elevation (70% of study cohort), those with only early elevation (measured during first three days following administration) and those with both early and late elevation (measured at 1 month after administration). Patients from the first group had no significant LVEF reduction and a very low incidence of cardiac events during a mean follow up of 20+/-13 months (the negative predictive value for CAE of normal TnI level was 99%) whereas nearly all patients from the second and third subgroups had LVEF reduction and a high frequency of CAE (respectively 37% and 84%, the most frequent being HF) (31).

More recently, Demissei et al. published the largest prospective study to date assessing clinical data, echocardiography and cardiac biomarkers in breast cancer patients receiving cardiotoxic agents (namely doxorubicin, cyclophosphamide and/or trastuzumab) with an extended follow-up period of 3.7 years (33). They found that elevated high sensitivity cardiac troponin T (hs-cTnT) levels were common after anthracycline therapy and their elevation at the completion of therapy, in contrast with serial measurement during chemotherapy, may be predictive of cancer therapy-related cardiac dysfunction (CTRCD). Moreover, changes in NT-pro-BNP were significantly associated with changes in LVEF and CTRCD occurrence and provided incremental value to baseline clinical risk factors for the prediction of CTRCD. Interestingly, preceding increases in NT-pro-BNP were associated with subsequent LVEF decline and altered circumferential strain, suggesting the potential interest of serial measurement for early detection of cardiotoxicity especially that induced by doxorubicin followed by trastuzumab. Other cardiac biomarkers are being studied, with myeloperoxidase being the most promising for the prediction of anthracyclines induced cardiotoxicity (32, 33). Our review didn’t identify any publication mentioning the use of biomarkers for prognostic assessment or cardiotoxicity detection during Tbt therapy.

Our study has several limitations. First, its retrospective nature and the heterogeneity of CAE reporting among clinical studies, may result in underestimation of the real incidence of Tbt-induced CAE. Second, stating a definite causal relationship between Tbt-administration and the onset of HF is difficult given the large number of confounding factors involved in cancer patients.

In conclusion, our systematic literature review suggested a Tbt-induced CAE incidence of 3.4% Data from more recent clinical trials and case reports highlight a higher rate of heart failure than previously reported as well as a possible interaction with anthracyclines. Data concerning the cardiac outcome is lacking but as suggested by our case and some others, HF could be completely reversible after drug discontinuation. Clinicians should be aware of such Tbt-associated acute cardiac toxicity and consider the risk-benefit ratio in patients with pre-existing cardiovascular disease. We suggest a possible interest of NT-pro-BNP for the early recognition of Tbt-associated heart failure.

## Author Contributions

JC, CJ, and BG managed the case and wrote the manuscript. VD performed the initial literature search and provided full text articles. CD performed database toxicologic research. All authors were involved in development of the manuscript, revising it critically for important intellectual content. All authors contributed to the article and approved the submitted version.

## Conflict of Interest

CJ received travel grants from PharmaMar S.A.

The remaining authors declare that the research was conducted in the absence of any commercial or financial relationships that could be construed as a potential conflict of interest.

## Publisher’s Note

All claims expressed in this article are solely those of the authors and do not necessarily represent those of their affiliated organizations, or those of the publisher, the editors and the reviewers. Any product that may be evaluated in this article, or claim that may be made by its manufacturer, is not guaranteed or endorsed by the publisher.

## Appendix 1

Equations for literature search

## Medline *via* OvidSP

(Trabectedin/OR Trabectedin.ti,ab,kw,nm OR Yondelis.ti,ab,kw,nm) AND (exp Heart Failure/OR Heart Failure*.ti,ab,kw OR Cardiac Failure*.ti,ab,kw OR Myocardial Failure*.ti,ab,kw OR Heart Decompensation*.ti,ab,kw OR cardiac edema*.ti,ab,kw OR ventricular dysfunction*.ti,ab,kw OR pulmonary edema*.ti,ab,kw OR diastolic dysfunction*.ti,ab,kw OR HFrEF.ti,ab,kw OR HRpEF.ti,ab,kw OR exp Cardiotoxicity/

OR exp Hypertension, Pulmonary/OR exp Natriuretic Peptides/OR cardiotoxicit*.ti,ab,kw OR cardio-toxicit*.ti,ab,kw OR Cardiac Toxicit*.ti,ab,kw OR cardio-oncolog*.ti,ab,kw OR cardiooncolog*.ti,ab,kw OR pulmonary hypertension*.ti,ab,kw OR natriuretic peptide*.ti,ab,kw OR Atrial Natriuretic Factor*.ti,ab,kw OR Auriculin.ti,ab,kw OR Atriopeptin*.ti,ab,kw OR Cardionatrin.ti,ab,kw OR Cardiodilatin.ti,ab,kw OR Atriopeptigen.ti,ab,kw)

## Scopus

TITLE-ABS-KEY [(Trabectedin OR Yondelis) AND (“Heart Failure” OR “Heart Failures” OR “Cardiac Failure” OR “Cardiac Failures” OR “insuffisance cardiaque” OR “insuffisances cardiaques” OR “Défaillance cardiaque” OR “Défaillances cardiaques” OR “Décompensation cardiaque” OR “Défaillance myocardique” OR “Défaillances myocardiques” OR “Myocardial Failure” OR “Myocardial Failures” OR “Heart Decompensation” OR “Heart Decompensations” OR “cardiac edema” OR “cardiac edemas” OR “oedème cardiaque” OR “oedèmes cardiaques” OR “ventricular dysfunction” OR “ventricular dysfunctions” OR “dysfonction ventriculaire” OR “dysfonctions ventriculaires” OR “pulmonary edema” OR “pulmonary edemas” OR “oedème pulmonaire” OR “oedèmes pulmonaires” OR “diastolic dysfunction” OR “diastolic dysfunctions” OR “dysfonction diastolique” OR “dysfonctions diastoliques” OR HFrEF OR HRpEF OR cardiotoxicit* OR cardio-toxicit* OR “Cardiac Toxicity” OR “Cardiac Toxicities” OR “Toxicité cardiaque” OR “Toxicités cardiaques” OR cardio-oncolog* OR cardiooncolog* OR “pulmonary hypertension” OR “pulmonary hypertensions” OR “hypertension pulmonaire” OR “hypertensions pulmonaires” OR “Cardiaques noirs” OR “maladie d’Ayerza” OR “syndrome d’Ayerza” OR “natriuretic peptide” OR “natriuretic peptides” OR “peptides natriurétiques” OR “Atrial Natriuretic Factor” OR “Atrial Natriuretic Factors” OR “facteur atrial natriurétique” OR “Facteur auriculaire natriurétique” OR Auriculin OR Atriopeptin* OR Cardionatrin OR Cardiodilatin* OR Atriopeptigen)]
